# When ontogeny recapitulates phylogeny: Fixed neurodevelopmental sequence of manipulative skills among primates

**DOI:** 10.1126/sciadv.abb4685

**Published:** 2020-07-24

**Authors:** Sandra A. Heldstab, Karin Isler, Caroline Schuppli, Carel P. van Schaik

**Affiliations:** Department of Anthropology, University of Zurich, Winterthurerstrasse 190, 8057 Zurich, Switzerland.

## Abstract

Neural development is highly conserved across distantly related species of different brain sizes. Here, we show that the development of manipulative complexity is equally cumulative across 36 primate species and also that its ontogeny recapitulates phylogeny. Furthermore, larger-brained species reach their adult skill levels later than smaller-brained ones, largely because they start later with the simplest techniques. These findings demonstrate that these motor behaviors are not modular and that their slow development may constrain their evolution. Complex foraging techniques therefore critically require a slow life history with low mortality, which explains the limited taxonomic distribution of flexible tool use and the unique elaboration of human technology.

## Teaser

The strictly cumulative emergence of manipulative skills in primates constrains the evolution of complex technology.

## INTRODUCTION

One of the most notable regularities of brain development in mammals is the highly conserved order of developmental events within and across species (*1*, *2*). This implies that the order of the emergence of traits is strictly cumulative: The presence of a particular trait is a necessary precondition for the emergence of another trait. The same finding also explains the tight relationship between the duration of neurodevelopment and adult brain size (*1*, *2*). However, the pattern documented in this previous work was largely restricted to the development of the central and peripheral nervous system, whereas behaviorally it only involved basic landmarks, such as reflexes or basic locomotor skills [see also (*3*)]. The question therefore arises whether this fixed developmental sequence across species also extends to more complex motor patterns with substantial fitness benefits, in particular manipulative skills of the forelimbs, and whether the duration of these aspects of development is likewise related to a species’ brain size. If the development of such skills also strictly requires the presence of specific preexisting underlying skills, this has important implications for the duration of development and thus for the kinds of species that could evolve the most complex manipulation skills needed for flexible tool use (independent use of hand and fingers, acting upon multiple objects). If, instead, these skills can be acquired independently, they become more or less modular, and selection can act independently of developmental constraints.

To properly test the stability in the developmental sequence of motor skills, we need longitudinal comparisons that comprise many species, cover the full manipulation spectrum, and use the same methods. Primates are especially suited for this because they use their hands in a great variety of food manipulations varying in form and complexity, including in tool use. Furthermore, complex manipulative skills in primates are expected to translate into improved diet quality and a more stable energy intake throughout the year and therefore to be directly related to fitness (*4*, *5*). However, most existing primate studies examining the ontogeny of manipulative skills were focused on single species and only a few manipulation types [e.g., (*6*–*11*)]. Other studies were conducted cross-sectionally [e.g., (*12*, *13*)], hampering the assessment of age-related changes. Last, because tool use acquisition has been the main focus of primate research, most work has concentrated on humans, macaques, capuchins, and great apes. Thus, so far, it has been impossible to conduct meaningful comparative tests of the prediction that complex manipulative skills are likewise strictly cumulative and have their timing linked to brain size.

Here, we follow a simple, but novel, approach that allows recognizing equivalent maturation states of the ontogeny of manipulative skills of hands and fingers across a broad variety of captive primates, encompassing 5 Strepsirrhini, 15 Platyrrhini, 10 Cercopithecoidea, and 6 Hominoid species (table S1). Using a consistent methodology and a mixed longitudinal–cross-sectional study design, including more than 10,000 observation bouts collected over more than 7 years, with interobservation intervals of maximally 2 months on specific individuals, we investigated the complete ontogenetic development of manipulative skills from the first week of birth until individuals achieved adult-level skill competence in these 36 primate species, which vary widely in developmental rates and life history strategies.

Our overall goal was to determine the ontogenetic order of emergence of eight different manipulative skills and to test whether the order is consistently cumulative or whether the most complex skills such as those required for tool manufacture and use can emerge at any time and are therefore modular instead. Likewise, by comparing our results with the interspecific comparison of adult manipulation complexity (*14*), which showed that species differences in maximum manipulation complexity were also cumulative, we test whether here, in contrast to the generally accepted notion, ontogeny recapitulates phylogeny: the hallmark of nonmodularity.

Concerning the developmental stage at which adult-level skill competence in food manipulations is reached, we test two additional predictions. First, as most primates need to be nutritionally self-supporting immediately following the weaning period due to the lack of postweaning provisioning (*15*), we predict that overall most subjects may reach adult-level manipulation complexity before or around weaning, but not after. Second, following from the finding of Workman *et al*. (*1*) and others [e.g., (*16*, *17*)] that larger-brained species have a relatively longer period of immaturity than smaller-brained species, we expect that larger-brained species will thus reach adult-level skill competence later, because they either start later, must go through more steps, or go through those steps more slowly. If these tests are confirmed, this strongly suggests that development of food-harvesting and processing skills constrains the duration of development, with interesting consequences for the coevolution between brain size and life history.

## RESULTS

### Ontogenetic order of emergence of manipulation categories

To assess whether food manipulations follow specific skill developmental trajectories, we repeatedly sampled 128 captive individuals of 36 species during feeding behavior over a period of more than 7 years. For 29 of these observed primate species, we were able to determine the exact age at which each of the eight manipulation complexity categories assigned before (*14*) was performed for the first time. These categories were based on all possible combination of the following: (i) use of the forelimbs, subdivided into unimanual and bimanual actions; (ii) asynchronous and synchronous use of hands; (iii) dependent or independent finger use; and (iv) for bimanual actions, whether the hands manipulated one object or multiple objects. To examine whether the order of emergence of manipulative skills is consistent across primate species and therefore truly cumulative across species, we used the deterministic Guttman scaling method (*18*). Manipulation categories are cumulative if an individual is able to perform a particular manipulation complexity category at age *N* only if it is also able to perform all lower-ranked manipulation complexity categories at age < *N*.

**Fig. 1:**
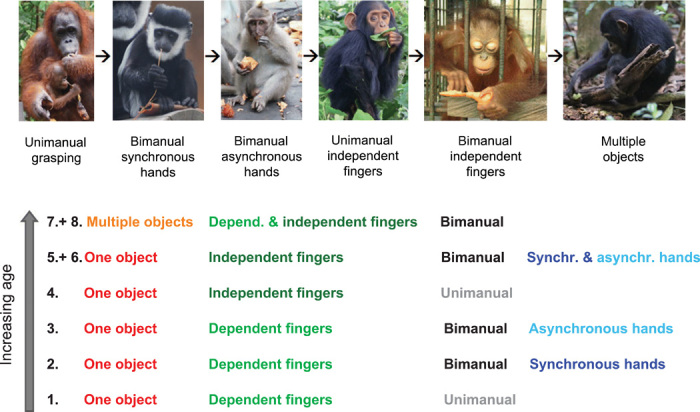
Eight food manipulation categories and their order of emergence during ontogeny. Ninety-seven percent of all observed species (*N* = 36) and 82% of all observed individuals (*N* = 128) strictly followed this ontogenetic sequence. As manipulation categories 5 and 6 and categories 7 and 8 emerged at the same time during ontogeny, each of these pairs of categories were merged into one new category (categories V and VI, respectively), resulting in six broad food manipulation categories for subsequent analyses. Species values are listed in table S1. Photo credit: S. A. Heldstab, University of Zurich; macaque picture: Marlen Fröhlich, University of Zurich; second orangutan picture: Zaida K. Kosonen, University of Zurich; chimpanzee pictures: Marlen Fröhlich, University of Zurich and Liran Samuni, Taï Chimpanzee Project.

Although individuals and species varied substantially in the timing of appearance and frequency of different manipulative skills, the order of emergence of the food manipulation categories during ontogeny was consistent within all but one, the silvery gibbon (*Hylobates moloch*), of the 36 observed primate species. In total, 97% of species’ performances and 82% of individuals’ performances exactly fitted the resulting Guttman scale, and the coefficient of reproducibility was close to 1 (0.93), indicating that our food manipulation categories can be placed in a cumulative rank order across species. The Guttman scale revealed the following ontogenetic order of emergence of food manipulation categories: First, individuals developed unimanual grasping of a single object with dependent finger use. This was followed by bimanual manipulations with dependent fingers and synchronous hands. Later, the capability to perform actions with dependent fingers and asynchronous hands emerged. Next, unimanual actions with independent fingers appeared, followed by bimanual actions with independent fingers. Multiple-object manipulations were the least to emerge (Fig. 1).

**Fig. 2:**
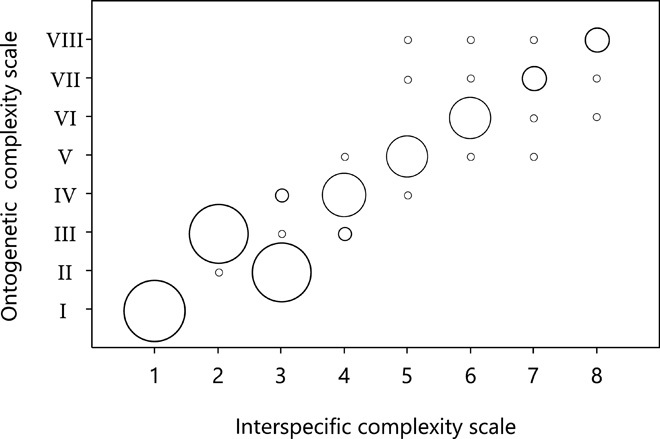
Relationship between the ontogenetic complexity scale and the interspecific complexity scale. The order of emergence of food manipulation categories during ontogeny follows almost exactly the order of the complexity scale found in the previous cross-species study of adults (*14*). The size of the circles indicates the number of species for the respective manipulation categories (see also fig. S2). The large variation of ontogenetic complexity categories in the upper right corner of the figure indicates that manipulation categories 5 and 6 and categories 7 and 8 emerged at around the same time during ontogeny and were therefore merged into one new category for subsequent analyses (see also Fig. 1).

This order of emergence of food manipulation categories during ontogeny follows almost exactly the order of the complexity scale found in the previous cross-species study of adults (Fig. 2 and fig. S2) (*14*). The few small differences between the ontogenetic and the interspecific complexity scale all arose because adjacent steps were switched. Thus, first, categories 2 and 3 changed their order so that individuals first developed bimanual manipulations with dependent fingers and synchronous hands and only later the capability to perform actions with dependent fingers and asynchronous hands. The second change relative to the interspecific scale was that categories 5 and 6, manipulating a single object with independent fingers and synchronous or asynchronous hands, developed roughly at the same time during ontogeny (Fig. 1). The same is also true for categories 7 and 8, bimanual multi-object manipulations with dependent or independent finger use, which also emerged together during ontogeny (Fig. 1). In conclusion, ontogeny broadly recapitulates phylogeny when it comes to object manipulation in primates.

### Age at adult-level skill competence

Age at adult-level skill competence was defined as the mean age at which immatures of a particular species were first observed to perform all food manipulation complexity categories of adult conspecifics assessed in our previous study (*14*). This age was then related to literature data on a species’ weaning age and brain size.

**Fig. 3:**
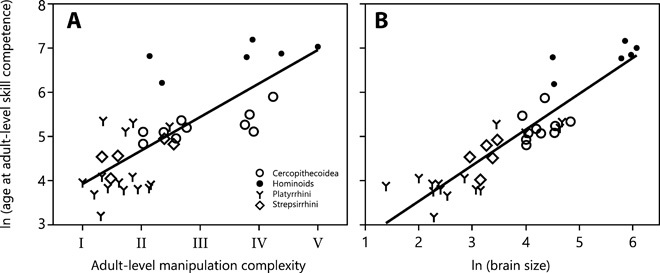
The age at which adult-level skill competence in food manipulations is reached is highly correlated with the complexity of the adult manipulation repertoire and with adult brain size. Relationship between age at adult-level skill competence [in ln (days)] and (**A**) the complexity of the adult manipulation repertoire and (**B**) adult brain size [in ln (cm^3^)] for 36 primate species. Skill competence in food manipulation is reached later in species with a more complex adult manipulation repertoire and with a larger adult brain size. Species values are listed in table S1.

**Fig. 4:**
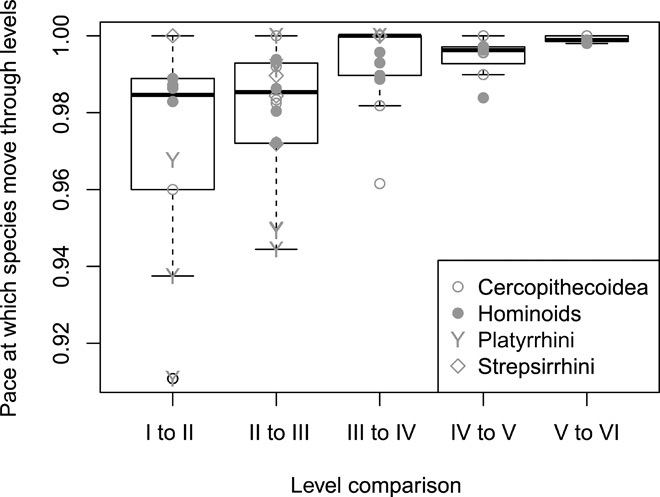
The pace at which individuals move from one level to the next adjacent level [approximated by the random slope estimates of the function level ~ log (age) with species as a random factor] is slower the higher the manipulation complexity level. Therefore, young primates are relatively fast in learning the simplest food manipulations, whereas there is a longer time lag between manipulation categories of higher complexity.

**Fig. 5:**
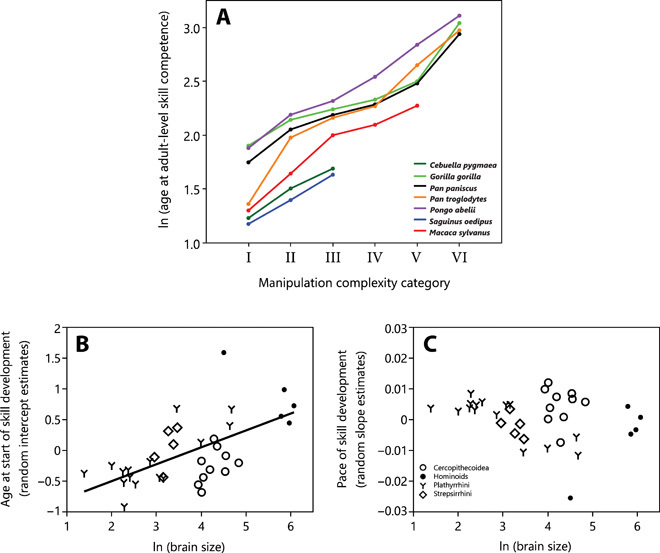
Immatures in large-brained species with a more complex manipulation repertoire need more time to develop their adult-level skill competence because they are older when they reach the first, simplest skill level. (**A**) Example of seven species illustrating the different starting points but the same pace of the development of food manipulation. (**B**) Larger-brained primate species start at an older age to manipulate food items, as shown by the relationship between brain size (log-transformed) and the estimated age at which infants of a given species first engage in unimanual grasping [approximated by random intercept estimates of the function log (age) ~ level with species as a random factor]. (**C**) Larger-brained primates do not transition more slowly to higher levels of manipulation complexity, as shown by the relationship between brain size (log-transformed) and the pace at which species move through the series of cumulative motor skills [approximated by the random slope estimates of the function level ~ log (age) with species as a random factor]. The notable hominoid outlier is the pileated gibbon (*H. pileatus*). The silvery gibbon (*H. moloch*) was excluded because it did not follow the order of emergence of the food manipulation categories during ontogeny of all the other species.

Overall, in most of the 36 species (94%), individuals reached adult-level manipulation complexity before or around weaning (fig. S3). The two exceptions were white-faced sakis (*Pithecia pithecia*) and pileated gibbons (*Hylobates pileatus*). Skill competence in food manipulation is reached later in species with a more complex adult manipulation repertoire [phylogenetic generalized least-squares regressions (PGLS): *P* = 0.043, λ = 0.985, *r*^2^ = 0.089, estimate = 0.233, SE = 0.111; Fig. 3A] and with a larger adult brain size (PGLS: *P* < 0.001, λ = 0.905, *r*^2^ = 0.430, estimate = 0.607, SE = 0.116; Fig. 3B). The pace at which individuals move from one level to the next adjacent level is slower the higher the level of complexity [linear least squares regressions (LM): *P* < 0.001, estimate = 0.007, SE = 0.002; Fig. 4] such that it takes longer to master the independent movement of fingers and hands or the handling of multiple objects than the simple grasping of items with one or two hands. Thus, as the feeding niche becomes more complex, maturing primates need more time to learn their food handling skills. Furthermore, with increasing brain size, species are already older when they first start to develop the different manipulation skills (PGLS: *P* = 0.024, λ = 0.989, *r*^2^ = 0.123, estimate = 0.291, SE = 0.123; Fig. 5, A and B). However, adult brain size was not correlated with the pace at which species move through the different levels (PGLS: *P* = 0.291, λ = 0.958, *r*^2^ = 0.005, estimate = −0.002, SE = 0.002; Fig. 5, A and C).

## DISCUSSION

### Ontogenetic order of emergence of manipulation categories

The fixed sequence for the development of the central and peripheral nervous system, basic reflexes, and rudimentary locomotor skills across mammals reveals that the development of brain functions is highly structured, i.e., cumulative and conserved across species (*1*). Here, we tested whether this law-like recapitulation also held for complex motor behaviors with substantial fitness benefits, manipulation of food, which obviously depend on brains for their fine motor control.

We found a marked extension of Workman’s pattern for motor actions (*1*): The ontogenetic order of emergence of six different manipulative skills was preserved across all 36 primate species despite large species differences in the duration of development. Some of this order is necessary and unremarkable, for example, that unimanual grasping precedes object manipulation. Unexpectedly, however, the order of all other manipulative skills appears to be equally conserved, suggesting that more complex motor skills also critically rely on particular preexisting skills.

Individuals first develop voluntary unimanual grasping of a single object with dependent fingers. This early manipulative activity emerges when infants first reach out while still clinging to the mother or another caregiver (*6*, *7*) after they have become able to overcome the reflexive closure of the entire hand, which is present at birth (*8*). This unimanual grasping is followed by bimanual manipulations with dependent fingers and synchronous hands and later by the ability to perform actions with asynchronous hands. Previous studies acknowledged that manipulations where both hands perform the same action are more straightforward for the brain to control than patterns where both hands perform different actions (*19*, *20*). This might be the reason for the earlier ontogenetic appearance of manipulation with synchronous hand instead of asynchronous hand use in our study. The emergence of these bimanual manipulations coincides with the diminution of clinging to the mother for postural support and the appearance of sitting on large branches or on the ground and independent locomotion (*6*, *13*), probably because two-handed actions require the body to be secured by clinging with the feet only, which is a mechanically more difficult task than clinging at three or four points.

Next, unimanual actions with independent finger use appear, followed by bimanual actions with independent fingers. Independent finger use is known to emerge relatively late during motor development due to neural maturation as it reflects the degree to which motor neurons innervating muscles acting on hands and fingers receive direct monosynaptic projections from the cerebral cortex. For instance, in rhesus macaques (*Macaca mulatta*), this occurs at the earliest at 2 to 4 months after birth, and mature independent finger use follows at least 4 months later (*7*, *21*). Multiple-object manipulation emerges last, which is in accordance with ontogenetic single-species studies in chimpanzees, capuchins, and macaques, showing that infants start to manipulate single objects, whereas manipulation of multiple objects, including tool use, emerged at later ages (*9*, *10* and references therein, *11*).

The intraspecific order of emergence of these six different motor skills during ontogeny follows almost exactly the interspecific order of the complexity scale of the same motor skills across adults of 36 primate species we found in a previous study (*14*). Thus, the ontogeny of manipulative skills broadly recapitulates its phylogeny, with the intra- and interspecific scale being equally well preserved. This finding is consistent with research on the recovery from neuronal diseases such as strokes in humans, which found that specific features of brain function revert to those seen at an early stage of development, with the subsequent process of recovery resembling ontogeny in many ways and thus suggesting an extremely fixed order of motor skill development (*22*). The nearly perfect cumulative order of emergence in both ontogenetic and phylogenetic relations explains why the most complex behaviors are relatively rarely observed across species.

These observations were all made in captivity. Wild individuals are likely exposed to much greater variety of manipulative challenges that cannot be easily captured in captivity. Data of our previous study (*14*) demonstrate that at least the types of food (sometimes including chopped fruits or pellets) available to the primates under study did not limit their capacity for manipulations, suggesting that captive individuals also have the opportunity to show the full range of manipulations.

A species’ potential for complex manipulations is likely limited by its hand morphology. The silvery gibbon (*H. moloch*) was the only species in our study to deviate from the skill developmental trajectory of all other species. Also, the second gibbon species in our dataset, the pileated gibbon (*H. pileatus*), was a notable outlier (see Fig. 5). Gibbons are forelimb-dominated climbers whose primary locomotor mode, brachiation, is quite distinctive from other primates. It favors a long hand functioning as a grasping hook during suspension and/or climbing that is less well suited for manipulative functions due to the reduced thumb/hand relationship (*23*). As expected, these two gibbon species both had a lower manipulation complexity than expected for their brain size (*14*). It is thus likely that these morphological trade-offs between locomotion and manipulation in gibbons are also responsible for the deviations from the ontogenetic trajectory of the other primate families. Similar outliers have been reported for nonprimates (*1*, *3*) but are remarkably rare.

### Age at adult-level skill competence

Workman *et al*. (*1*) and Finlay and Darlington (*2*) showed that the developmental timing of appearance of basic behaviors such as reflexes or basic locomotor behaviors in their sample of 18 mammal species can be explained by a single factor, namely, eventual brain size [see also (*3*)]. Following up on this finding, we investigated whether adult brain size is also sufficient to predict the timing of emergence of more complex motor skills. We found that the age at which adult-level skill competence in food manipulations is reached is highly correlated with adult brain size. Furthermore, the present study also showed that the larger a species’ adult brain size, the longer the developmental duration until the full repertoire of food manipulations is learned, because large-brained species start to first manipulate food items at an older age rather than going through the series of cumulative motor skills at a slower pace. This pattern arises because infants of larger-brained species such as chimpanzees, especially humans, are born with far less mature brains compared to smaller-brained anthropoid primates (*16*), although our current sample is too small to test this statistically. In a previous study, we found that a species’ manipulation complexity coevolved with brain size (*14*), which is consistent with work (*24*) showing that the relative amount of cerebral cortex devoted to the corticospinal tract is related to digital dexterity. Our present results corroborate the suggestion (*1*, *25*) that selection for any trait associated with large brain size simultaneously means selection for a particular developmental duration, and vice versa. Thus, there will be developmental constraints on the evolution of the most complex manipulation skills, such as those for technology, which also take longer to acquire than the less complex skills.

During food manipulation learning periods, failures are common and net yields are low (*26*). Furthermore, large-brained species also need to overcome the high energetic costs of brain growth and maintenance during the same long time window (*27*). For a species to overcome these developmental costs and gain a fitness payoff by enhanced manipulative skills, e.g., to find or gain access to high-quality food items and avoid starvation, this species must be long-lived. But conversely, more skilled individuals also acquire more or better food and are better at avoiding starvation and thus survive longer. Thus, as suggested by the “needing-to-learn” hypothesis (*28*), complex manipulative skills and a slow life history with a prolonged developmental period and longer survival have coevolved in primates, as shown by the tight relationship between brain size and the duration of immaturity and adulthood (*16*, *17*).

The coevolution of food manipulation development and life history milestones is therefore also evident in the timing of weaning, which marks the end of parental energy subsidies. Because the consequences of food acquisition incompetence are especially severe during development, a juvenile’s foraging skills at weaning age must have reached a level sufficient to support their growing body. As expected, we found that most subjects reached adult-level manipulation complexity before or at weaning. Our results are consistent with previous studies showing that the competence in foraging skills roughly coincides with weaning in a variety of primate species (*12*, *26*, *29*–*31*). Although many other behavioral and physiological factors no doubt also played a role, primates’ foraging skill acquisition and the timing of weaning underwent strong coevolution.

In all four tool-using species of our dataset (bonobos, chimpanzees, gorillas, and orangutans), we observed all basic manipulations necessary for tool use before or around weaning. However, weaned immatures were never successful in obtaining food items during the first tool-using trials. Similarly, adult-level proficiency in tool use and feeding on hard-to-process food items requiring top-end complexity manipulation techniques in wild chimpanzees, orangutans, macaques, and capuchins is often reached only during late juvenility or even adulthood (*9*–*11*, *26*).

We have shown that the manipulative skills a species can acquire are strongly linked to its life history and selection for technological abilities such as tool use can therefore only be expressed in species with enough time to learn during development. We suggest that complex manipulative skills only evolve if they buy the species a strong increase in adult survival to compensate for the slowdown of development. These findings therefore imply that species with manufacture and flexible use of tools can only reach such levels of technology if they have enough time during ontogeny, suggesting that they need to have reached a sufficiently slow life history pace to permit such a change in their foraging niche. The evolution of the superb technological skills in the hominin lineage was therefore built on a slow life history and extended the coevolution of ecological skills, brain size, and life history that started in the hominoids (*16*, *17*, *32*–*34*).

## MATERIALS AND METHODS

### Behavioral observations

In total, we assessed longitudinal skill development of food manipulations in 128 captive individuals (on average, 4 individuals per species; range, 1 to 12 individuals per species) of 36 primate species (table S1). Data were collected in a similar manner as for the previous study of manipulation complexity (*14*), allowing for comparability between the newly collected data on immature individuals with the data pertained to the adults from the former study. Subjects were observed in their home enclosures, as previous work had shown that infant primates do not display the full extent of their manipulative abilities when observed out of their home environment (*6*).

Data were collected by behavioral sampling between October 2011 and November 2018, for a total of 762 hours in 13 different zoos and the primate station of the Department of Anthropology, University of Zurich (table S1). As in the previous study, manipulation was defined as making physical contact with a food item with the forelimbs and thus did not include visual exploration or sniffing without contact (*14*). Behavioral sampling was conducted over bouts of maximally 5 min. This duration has been shown to be sufficient, as object manipulation bouts in free-living immature individuals have been shown to be of a mean length of less than 2 to 4 min (*35*, *36*). The observation began as soon as the subject started to manipulate a food item and ended when contact was terminated, or after 5 min. After each individual observation bout, there was an interval of at least 2 min without sampling to decrease autocorrelation. A minimum of 20 bouts per individual per observation period were collected. In a previous study, we demonstrated that 20 bouts suffice to evaluate the full potential of manipulation complexity by constructing so-called collector’s (saturation) curves (*14*). We started to observe immatures during the first week after birth and continued behavioral sampling at regular intervals (every single week to 2 months, depending on the species’ life history) until individuals achieved adult-level manipulative skill competence.

In total, 10,936 bouts were recorded. For every behavioral bout, we investigated which of the eight manipulation categories assigned in the previous study (*14*) were performed by the subject under observation. These categories were based on all possible combination of the following: (i) use of the forelimbs, subdivided into unimanual and bimanual actions; (ii) asynchronous and synchronous use of hands; (iii) dependent or independent finger use; (iv) and in bimanual actions, we distinguished between the hands manipulating one object or multiple objects. We only scored the presence of manipulation categories if the observed individual performed a manipulation category at least twice. Frequency or duration of use of manipulation categories was not assessed.

### Ontogenetic order of emergence of manipulation categories

For 29 of the 36 observed primate species, we were able to determine the exact age at which each food manipulation category emerged during ontogenetic development (see below). This allows us to examine whether the order of emergence of manipulative skills is consistent across primate species. To do this, we used the deterministic Guttman scaling method (*18*) based on the description of Green (*37*). Using Guttman’s scaling method (*18*), we can test whether the order of emergence of particular skills during ontogeny (in our case, of food manipulation categories) is truly cumulative across species. Manipulation categories are cumulative if an individual is able to perform a particular manipulation complexity category at age *N* only if it is also able to perform all lower-ranked manipulation complexity categories at age < *N*. For any empirical set of observed skills, the coefficient of reproducibility indicates the extent to which the order of learned skills does fit such a cumulative scale and hence determine the percentage of species and/or individuals that follow the same order of emergence of manipulation categories.

### Age at adult-level skill competence

To investigate at which age young primates reach adult-level food manipulation competence, age at adult-level skill competence was defined as the mean age at which immatures of a particular species were able to perform all food manipulation complexity categories of adult conspecifics. The accuracy at which the age of skill competence could be determined varied from 1 week (i.e., *Callitrichidae*) to 2 months (i.e., great apes) depending on the species’ life history. Three categories of skill development trajectories were defined: (i) Adult level of food manipulation skills is reached before weaning: before 85% of the species’ average weaning age is completed; (ii) adult level of food manipulation skills is reached around weaning: after 85% and before 115% of the weaning age is completed; and (iii) all manipulation complexity categories are reached after weaning: after 115% of the developmental period until weaning is completed [following (*34*)]. Data on weaning age were taken from the literature (table S1) and verified with our own observations, where we examined during each observational period whether immatures were suckling. To test whether age at adult-level skill competence is influenced by adult brain size, data on adult-level manipulation complexity and endocranial volumes of mostly wild-derived female primates were retrieved from the literature (table S1).

### Statistical analyses

All statistical analyses and plots were performed using JMP 10.0 (*38*) and R 3.4.1 (*39*). The values of age at adult-level skill competence and adult brain size were log*_e_*-transformed to reach residuals that were evenly distributed around zero. To investigate the interspecific relationship between age at adult-level skill competence and adult brain size, PGLS (*40*) with the “caper” package (*41*) were used to control for phylogenetic nonindependence. Phylogeny was based on a composite supertree (fig. S1) (*42*). To investigate the effect of brain size on the age at which species develop the different manipulative skills and on the pace at which species move through the series of cumulative motor skills, we used a generalized linear mixed model [GLMM; package “lme4”: (*43*)], with age as a function of level (to reach a linear relationship, age had to be log-transformed) and species as a random factor, using a Gaussian family distribution. For each species, this generated a random intercept and a random slope. We used the random intercepts generated by the GLMM as an approximation of the age at which manipulative skills start to develop in the different species and the random slopes as an approximation of the pace at which the different species move through the different cumulative steps. In a next step, we performed two PGLS models to test the effect of brain size on these random intercepts and slopes, respectively. To compare the pace at which species move from one level to the next as a function of level complexity, for each species and for each adjacent level (L1 to L2, L2 to L3, L3 to L4, L4 to L5, and L5 to L6), we computed the estimates of the level as a function of age using LM (package “stats”). We then used an LM model to test for the effect of the level complexity on these estimates representing the pace at which species move from one level to the next.

## Supplementary Material

abb4685_SM.pdf

abb4685_Table_S1.xlsx

## SUPPLEMENTARY MATERIALS

Supplementary material for this article is available at http://advances.sciencemag.org/cgi/content/full/6/30/eabb4685/DC1

## Appendix

View/request a protocol for this paper from *Bio-protocol*.
