# Supplementary material for: When ontogeny recapitulates phylogeny: Fixed neurodevelopmental sequence of manipulative skills among primates
Source: Sci Adv. 2020 Jul 24;6(30):eabb4685. doi: 10.1126/sciadv.abb4685 (PMC7380958; doi:10.1126/sciadv.abb4685)
Supplement: abb4685_SM.pdf [file abb4685_SM.pdf]

Science Advances
AAAS

advances.sciencemag.org/cgi/content/full/6/30/eabb4685/DC1

Supplementary Materials for

When ontogeny recapitulates phylogeny: Fixed neurodevelopmental sequence of manipulative skills among primates

Sandra A. Heldstab*, Karin Isler, Caroline Schuppli, Carel P. van Schaik

*Corresponding author. Email: sandra.heldstab@uzh.ch

Published 24 July 2020, *Sci. Adv.* **6**, eabb4685 (2020)
DOI: 10.1126/sciadv.abb4685

The PDF file includes:

Figs. S1 to S3

Other Supplementary Material for this manuscript includes the following:

(available at advances.sciencemag.org/cgi/content/full/6/30/eabb4685/DC1)

Table S1

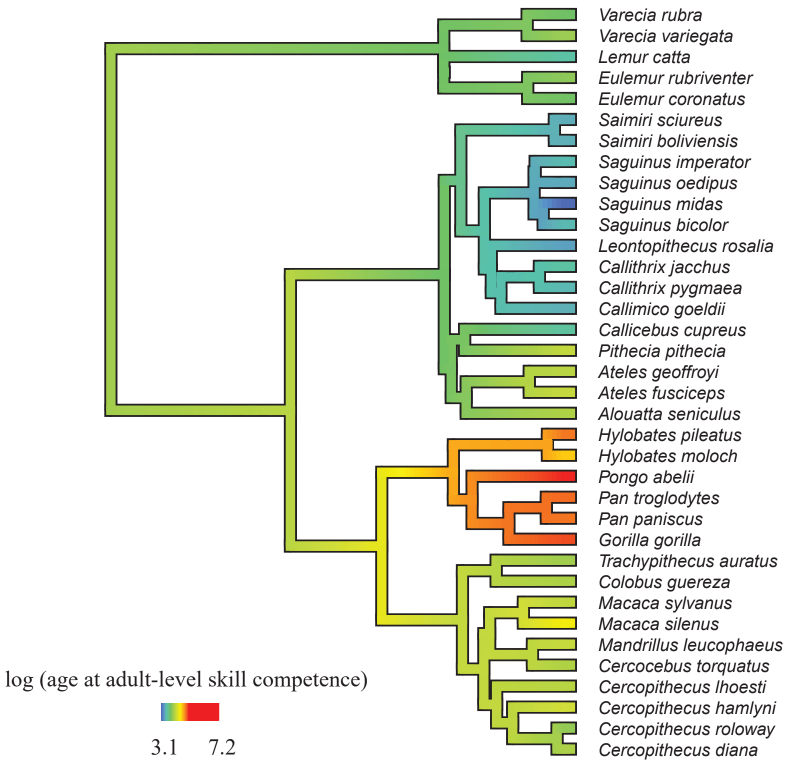

Fig. S1. The phylogenetic tree from (*42*) of 36 primate species used in the present study with an indication how logarithmized age at adult-level skill competence in days is distributed across the tree, ranging from very short (dark blue) to very long (red).

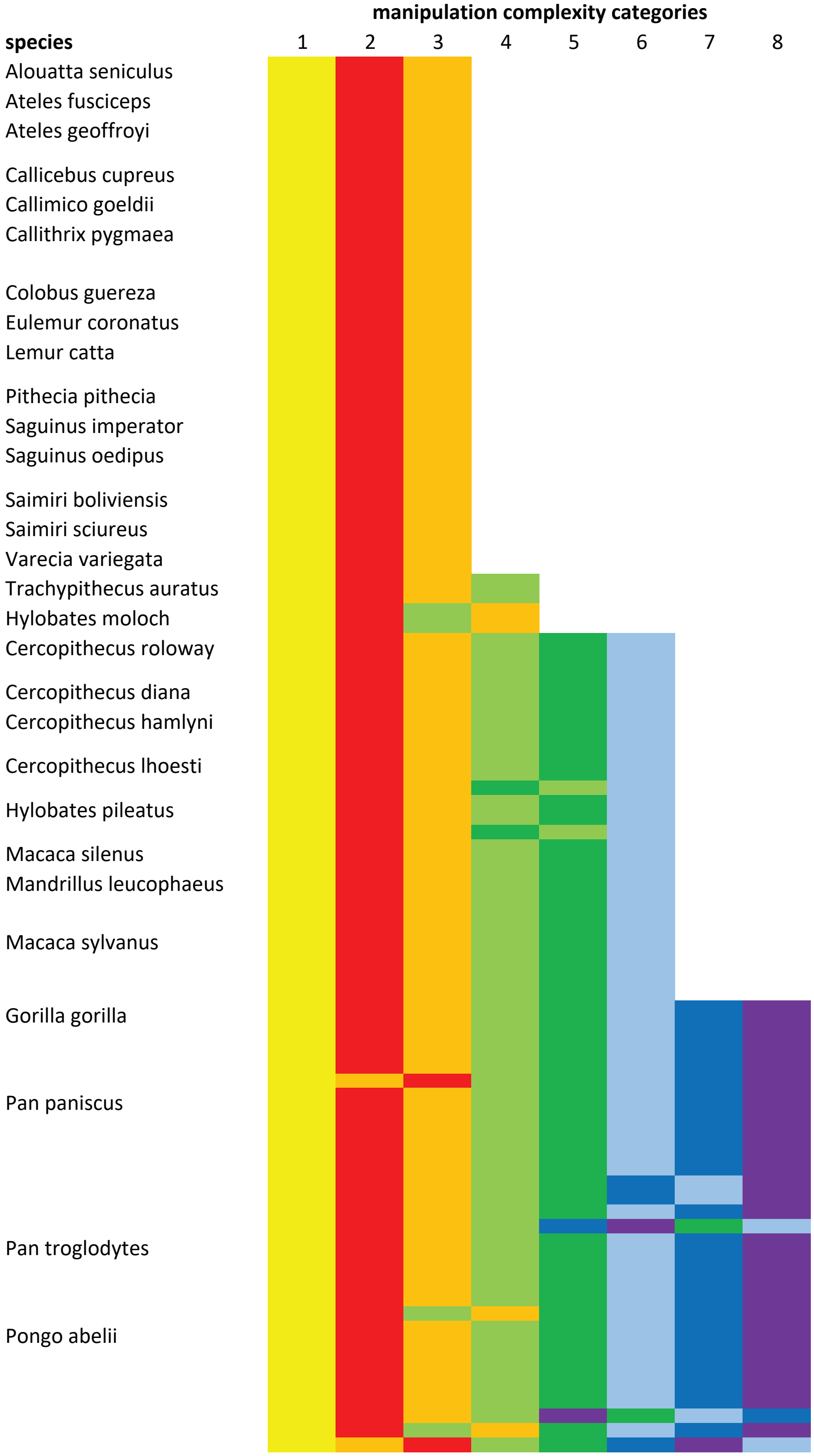

Fig. S2. Comparison between the interspecific manipulation complexity scale assigned in the previous study (*14*) and the ontogenetic scale of the present study with color coding. Each line represents one individual (between 1 and 9 individuals per species). The order of emergence of food manipulation categories during ontogeny follows almost exactly the order of the complexity scale found in the previous cross-species study. The few small differences between the ontogenetic and the interspecific complexity scale were all because adjacent steps were switched, e.g. categories 2 and 3 as well as categories 5 and 6.

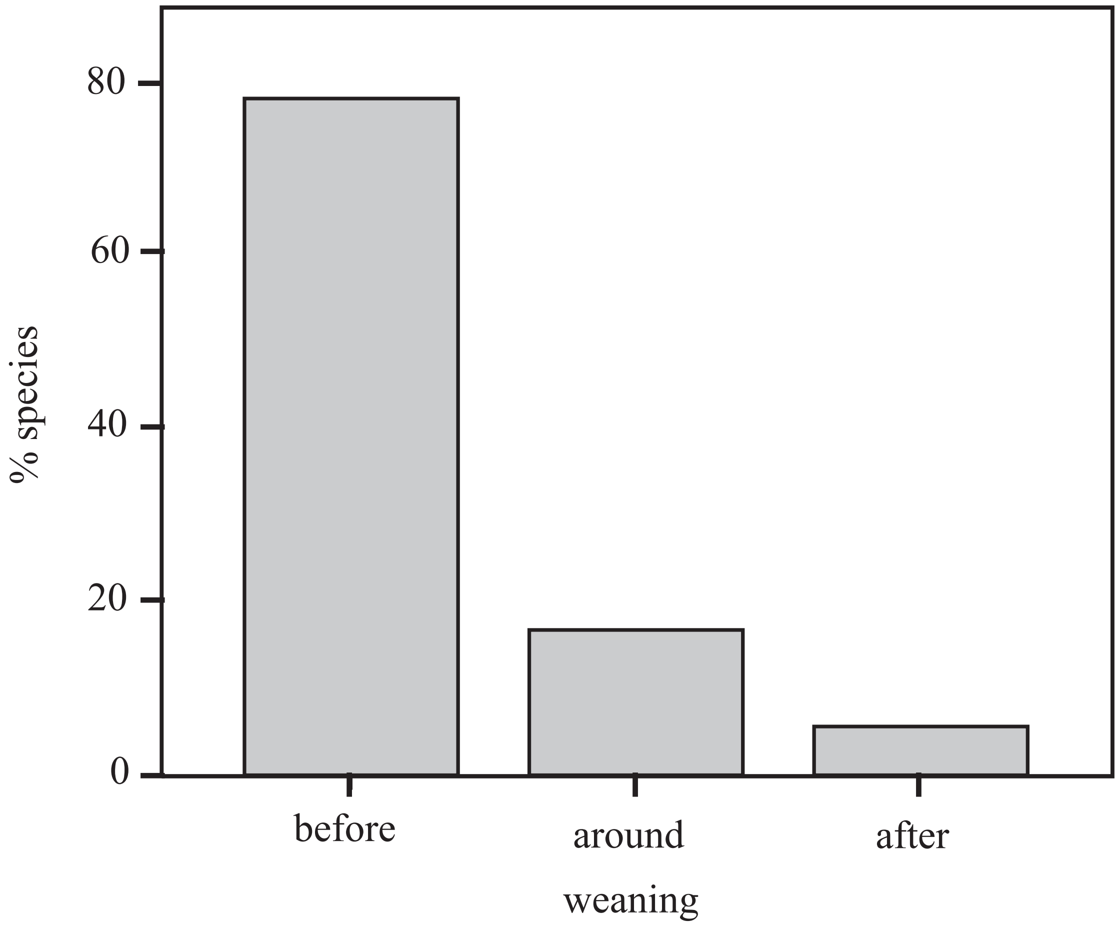

Fig. S3. Distribution of the different classes of food manipulation skill development in primates. Most out of a total of 128 individuals in 36 species reach adult-level food manipulation competence before or around weaning: 28 species, at weaning: 6 species, after weaning: 2 species). Species values are listed in Supplementary Materials Table S1.
